# Supplementary material for: An esophagus cell atlas reveals dynamic rewiring during active eosinophilic esophagitis and remission
Source: Nat Commun. 2024 Apr 18;15:3344. doi: 10.1038/s41467-024-47647-0 (PMC11026436; doi:10.1038/s41467-024-47647-0)
Supplement: Supplementary file 1 — Supplementary Information [file 41467_2024_47647_MOESM1_ESM.pdf]

Supplemental information for

An esophagus cell atlas reveals dynamic rewiring during active eosinophilic esophagitis and remission

Jiarui Ding[1,2,†], John J. Garber[3,4,†,*], Amiko Uchida[3], Ariel Lefkovith[1], Grace T. Carter[1], Praveen Vimalathas[3,4], Lauren Canha[3], Michael Dougan[3], Kyle Staller[3], Joseph Yarze[3], Toni M. Delorey[1], Orit Rozenblatt-Rosen[1,8], Orr Ashenberg[1], Daniel B. Graham[1,4,5,6], Jacques Deguine[1], Aviv Regev[1,7,8,*], Ramnik J. Xavier[1,4,5,6,*]

[1]Klarman Cell Observatory, Broad Institute of MIT and Harvard, Cambridge, MA 02142, USA
[2]Department of Computer Science, University of British Columbia, Vancouver, BC, V6T 1Z4, Canada
[3]Gastrointestinal Division, Department of Medicine, Massachusetts General Hospital, Boston, MA 02114, USA
[4]Center for the Study of Inflammatory Bowel Disease, Massachusetts General Hospital, Harvard Medical School, Boston, MA 02114, USA
[5]Center for Computational and Integrative Biology, Massachusetts General Hospital, Harvard Medical School, Boston, MA 02114, USA
[6]Department of Molecular Biology, Massachusetts General Hospital, Harvard Medical School, Boston, MA 02114, USA
[7]Department of Biology, Massachusetts Institute of Technology, Cambridge, MA 02142, USA
[8]Genentech, South San Francisco, CA 94080, USA
[†]These authors contributed equally: Jiarui Ding, John J. Garber
[*]Correspondence: garber.md@gmail.com (J.J.G.) , aviv.regev.sc@gmail.com (A.R.), xavier@molbio.mgh.harvard.edu (R.J.X.)

Supplementary **Fig**. 1 to **Fig**. 10
Supplementary Note 1
Supplementary References

Supplementary Figures

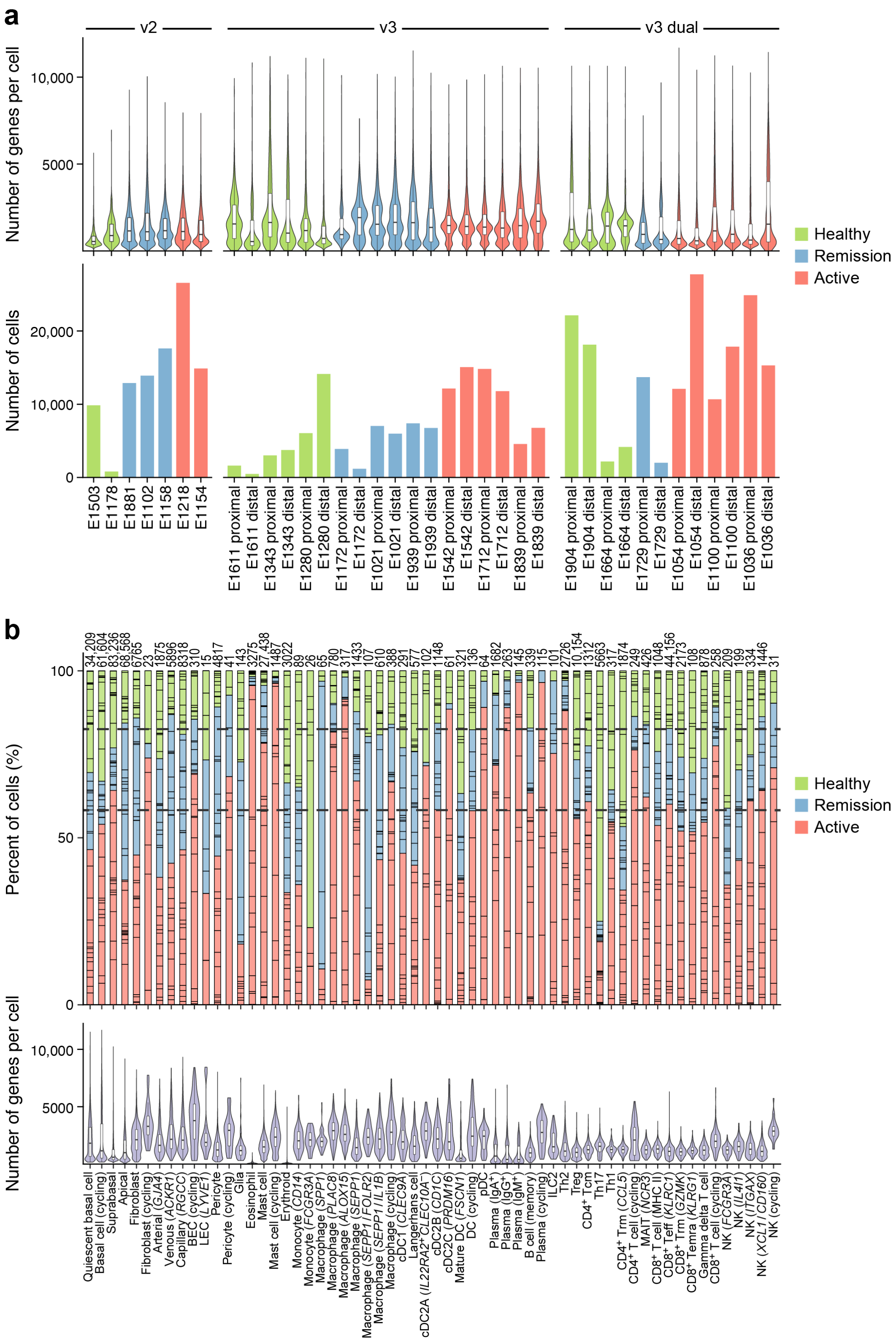

Supplementary Fig. 1. Quality control metrics of the esophageal scRNA-seq atlas. **a.** Distribution of the number of detected genes per cell (*y* axis, top) and the number of cells detected (*y* axis, bottom) in each biopsy (*x* axis) from healthy (*n* = 12, green), remission (*n* = 11, blue), and active EoE (*n* = 14, red). Data are grouped by 10x Chromium chemistry (v2, v3, and v3-dual index). **b.** Percent of cells (*y* axis, top) in each sample from each disease category (bar stacking and colors, top) and distribution of the number of genes per cell (*y* axis bottom) for each cell subset (*x* axis). The number of cells in each subset is labeled on top. Violin plot width: Gaussian kernel density estimation of data with default parameters, scaled to have a maximum of 1; White horizontal segment: median. Source data are provided as a Source Data file.

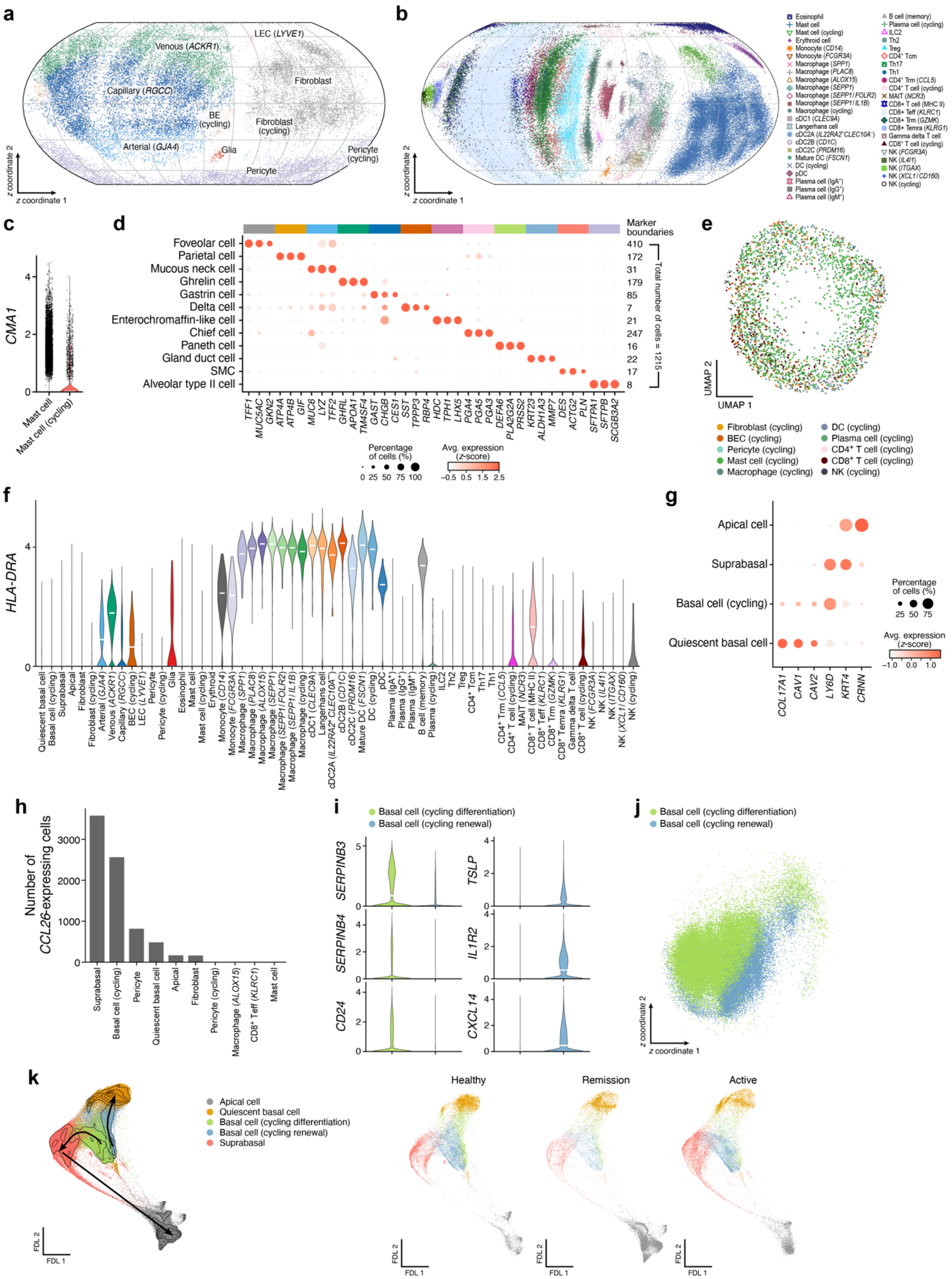
a
Venous (ACKR1)
LEC (LYVE1)
Fibroblast
Capillary (RGCC)
BE (cycling)
Fibroblast (cycling)
Arterial (GJA4)
Glia
Pericyte (cycling)
Pericyte
z coordinate 2
z coordinate 1
b
c
CMA1
Mast cell
Mast cell (cycling)
d
Marker boundaries
Foveolar cell
Parietal cell
Mucous neck cell
Ghrelin cell
Gastrin cell
Delta cell
Enterochromaffin-like cell
Chief cell
Paneth cell
Gland duct cell
SMC
Alveolar type II cell
Total number of cells = 1215
Percentage of cells (%)
Avg. expression (z-score)
e
UMAP 2
UMAP 1
Fibroblast (cycling)
BEC (cycling)
Pericyte (cycling)
Mast cell (cycling)
Macrophage (cycling)
DC (cycling)
Plasma cell (cycling)
CD4+ T cell (cycling)
CD8+ T cell (cycling)
NK (cycling)
f
HLA-DRA
g
Apical cell
Suprabasal
Basal cell (cycling)
Quiescent basal cell
COL17A1
CAV1
CAV2
LY6D
KRT4
CRNN
Percentage of cells (%)
Avg. expression (z-score)
h
Number of CCL26-expressing cells
Suprabasal
Basal cell (cycling)
Pericyte
Quiescent basal cell
Apical
Fibroblast
Pericyte (cycling)
Macrophage (ALOX15)
CD8+ Teff (KLRC1)
Mast cell
i
Basal cell (cycling differentiation)
Basal cell (cycling renewal)
SERPINB3
SERPINB4
CD24
TSLP
IL1R2
CXCL14
j
Basal cell (cycling differentiation)
Basal cell (cycling renewal)
z coordinate 2
z coordinate 1
k
Apical cell
Quiescent basal cell
Basal cell (cycling differentiation)
Basal cell (cycling renewal)
Suprabasal
Healthy
Remission
Active
FDL 2
FDL 1

Supplementary Fig. 2. scRNA-seq atlas of the human esophageal mucosa. **a,b.** Stromal and immune compartments. 2D spherical latent representation of stromal (**a**) and immune (**b**) cell profiles (dots) from all donors (n = 22), colored by cell type, as learned by scPhere, taking patient, disease status, and anatomical region of biopsy as batch factors. **c**. A small fraction of mast cells expresses *CMA1*. Distribution of expression (y axis, $\log_{10}$(TP10K+1)) of *CMA1* in mast cell subsets (x axis). **d**. Marker genes for 12 rare cell subsets. Mean expression (dot color; column Z-score) and proportion of expressing cells (dot size) of marker genes (columns) for each of the 12 rare subsets (rows) detected in one or a few patient biopsies. Number on right: total number of cells of a type. **e.** Cycling cells in the atlas. UMAP embedding of cell profiles (dots) from all cycling cells in the atlas (except cycling basal cells), colored by type, based only on expression of cell-cycle specific genes[1]. **f**. *HLA-DRA* expression is detected across cell types even after extensive removal of ambient RNAs. Distribution of expression (y axis, $\log_{10}$(TP10K+1)) of *HLA-DRA* in each cell subset (x axis). **g.** Validated esophageal epithelial marker genes[2] are expressed in the appropriate cell subsets. Mean expression (dot color) and proportion of expressing cells (dot size) for esophageal epithelial marker genes previously validated by immunofluoresence[2] (columns) across epithelial cell subsets in our atlas (rows). **h.** *CCL26* expressing subsets. Number of cells (y axis) expressing *CCL26* in the top 10 *CCL26*-expressing subsets based on the number of cells with greater than two detected *CCL26* transcripts (x axis). **i–k.** Basal cell differentiation. **i.** Cycling basal cells can be further divided into 'differentiation' and 'renewal' subtypes based on expression of suprabasal and basal cell marker genes. Distribution of expression (y axis, $\log_{10}$(TP10K+1)) of key marker genes distinguishing 'cycling differentiation' (green) and 'cycling renewal' (blue) basal cells (x axis). **j.** ScPhere embedding of cycling differentiation' (green) and 'cycling renewal' (blue) basal cell profiles. **k.** Force-directed layout of epithelial cell profiles colored by cell type. Arrows indicate putative cell differentializing trajectories. Violin plot widths: Gaussian kernel density estimation of data with default parameters, scaled to a maximum of 1; white horizontal segment: median. Source data are provided as a Source Data file.

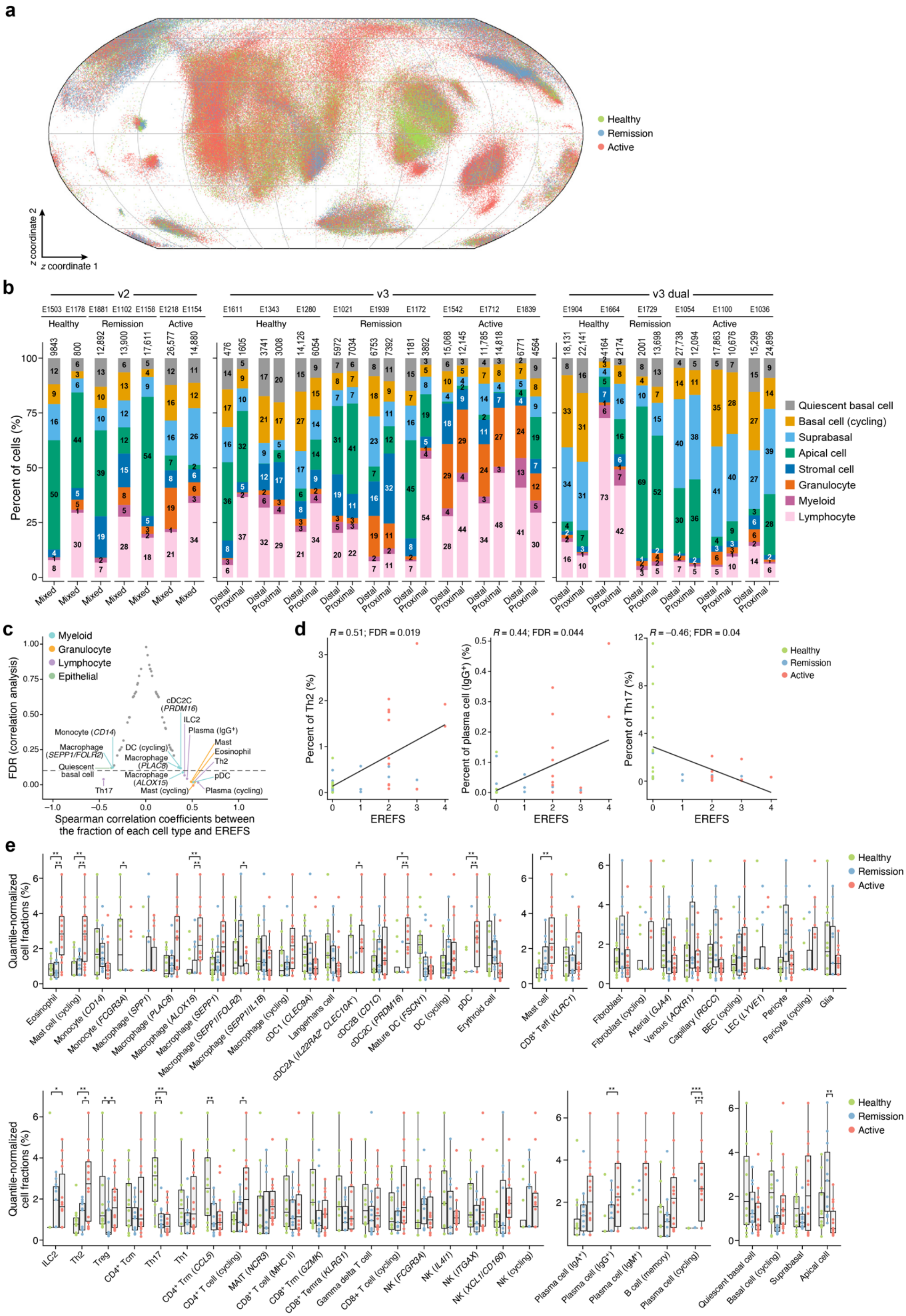

a
Healthy
Remission
Active
z coordinate 2
z coordinate 1
b
v2
v3
v3 dual
Healthy
Remission
Active
Percent of cells (%)
Quiescent basal cell
Basal cell (cycling)
Suprabasal
Apical cell
Stromal cell
Granulocyte
Myeloid
Lymphocyte
Mixed
Distal
Proximal
c
Myeloid
Granulocyte
Lymphocyte
Epithelial
FDR (correlation analysis)
Spearman correlation coefficients between the fraction of each cell type and EREFS
Monocyte (CD14)
Macrophage (SEPP1/FOLR2)
Quiescent basal cell
Th17
DC (cycling)
Macrophage (PLAC8)
Macrophage (ALOX15)
Mast (cycling)
cDC2C (PRDM16)
ILC2
Plasma (IgG+)
Mast
Eosinophil
Th2
pDC
Plasma (cycling)
d
R = 0.51; FDR = 0.019
R = 0.44; FDR = 0.044
R = −0.46; FDR = 0.04
Percent of Th2 (%)
Percent of plasma cell (IgG+) (%)
Percent of Th17 (%)
EREFS
Healthy
Remission
Active
e
Quantile-normalized cell fractions (%)
Healthy
Remission
Active
Eosinophil
Mast cell (cycling)
Monocyte (CD14)
Monocyte (FCGR3A)
Macrophage (SPP1)
Macrophage (PLAC8)
Macrophage (ALOX15)
Macrophage (SEPP1)
Macrophage (SEPP1/FOLR2)
Macrophage (SEPP1/IL1B)
Macrophage (cycling)
cDC1 (CLEC9A)
Langerhans cell
cDC2A (IL22RA2+ CLEC10A+)
cDC2B (CD1C)
cDC2C (PRDM16)
Mature DC (FSCN1)
DC (cycling)
pDC
Erythroid cell
Mast cell
CD8+ Teff (KLRC1)
Fibroblast
Fibroblast (cycling)
Arterial (GJA4)
Venous (ACKR1)
Capillary (RGCC)
BEC (cycling)
LEC (LYVE1)
Pericyte
Pericyte (cycling)
Glia
ILC2
Th2
Treg
CD4+ Tcm
Th17
Th1
CD4+ Trm (CCL5)
CD4+ T cell (cycling)
MAIT (NCR3)
CD8+ T cell (MHC II)
CD8+ Trm (GZMK)
CD8+ Temra (KLRG1)
Gamma delta T cell
CD8+ T cell (cycling)
NK (FCGR3A)
NK (IL4I1)
NK (ITGAX)
NK (XCL1/CD160)
NK (cycling)
Plasma cell (IgA+)
Plasma cell (IgG+)
Plasma cell (IgM+)
B cell (memory)
Plasma cell (cycling)
Quiescent basal cell
Basal cell (cycling)
Suprabasal
Apical cell

Supplementary Fig. 3. Cellular compositional shifts between active EoE, remission, and health. **a**. ScPhere embedding (as in Fig. 1c) of cell profiles (dot) colored by disease status. **b.** Percent of cells (*y* axis) of each major compartment (color code) in each biopsy (*x*-axis) ordered by 10x Chromium chemistry (v2, v3, and v3-dual index). Sample ID, disease status, and total number of detected cells are indicated on top. **c**,**d.** Cell proportion association with endoscopic reference score (EREFS). **c**. FDR (*y* axis) of Spearman rank correlation coefficients (*x* axis) between EREFS and the number of cells of each subset in each patient (n = 22). Cell types with absolute Spearman correlation coefficients > 0.35 are shown. Dashed horizontal line: FDR = 0.1, two-tailed one-sample Student's *t*-test. **d**. Percent cells of specific types (*y* axis) and EREFS (*x* axis) in each donor (dot). Lines: Linear regression. Two-tailed one-sample Student's *t*-tests. **e.** Changes in cell composition between conditions. Distributions of cell type proportions after quantile normalization (*y* axis) in active disease (red, n = 14), remission (blue, n = 11), or healthy (green, n = 12) biopsies (points). Boxplots: medians and interquartile ranges (IQR). Whiskers: lowest datum within 1.5 IQR of the lower quartile and highest datum within 1.5 IQR of the upper quartile. *** BH FDR < 0.001, ** FDR < 0.01, * FDR < 0.05, two-tailed Wald test. Source data are provided as a Source Data file.

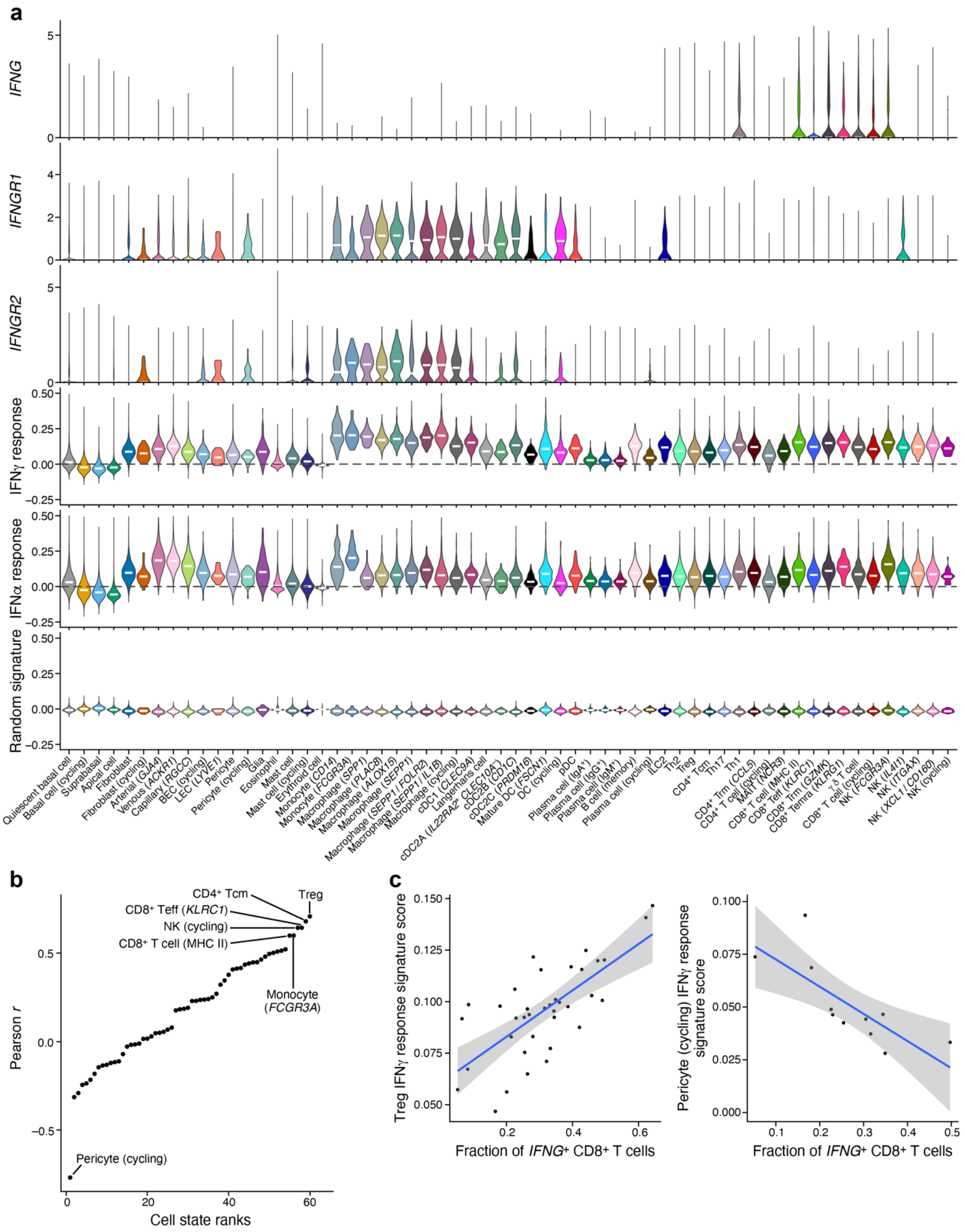

a
IFNG
IFNGR1
IFNGR2
IFNγ response
IFNα response
Random signature
Quiescent basal cell
Basal cell (cycling)
Suprabasal
Apical cell
Fibroblast
Fibroblast (cycling)
Arterial (GJA4)
Venous (ACKR1)
Capillary (RGCC)
BEC (cycling)
LEC (LYVE1)
Pericyte
Pericyte (cycling)
Glia
Eosinophil
Mast cell
Mast cell (cycling)
Erythroid cell
Monocyte (CD14)
Monocyte (FCGR3A)
Macrophage (SPP1)
Macrophage (PLAC8)
Macrophage (ALOX15)
Macrophage (SEPP1)
Macrophage (SEPP1/FOLR2)
Macrophage (SEPP1/IL1B)
Macrophage (cycling)
cDC1 (CLEC9A)
Langerhans cell
cDC2A (IL22RA2/CLEC10A)
cDC2B (CD1C)
cDC2C (PRDM16)
Mature DC (FSCN1)
DC (cycling)
pDC
Plasma cell (IgA+)
Plasma cell (IgG+)
Plasma cell (IgM+)
B cell (memory)
Plasma cell (cycling)
ILC2
Th2
Treg
CD4+ Tcm
Th17
Th1
CD4+ Trm (CCL5)
CD4+ T cell (cycling)
MAIT (NCR3)
CD8+ T cell (MHC II)
CD8+ Teff (KLRC1)
CD8+ Trm (GZMK)
CD8+ Temra (KLRG1)
γδ T cell
CD8+ T cell (cycling)
NK (FCGR3A)
NK (IL4I1)
NK (ITGAX)
NK (XCL1/CD160)
NK (cycling)
b
Pearson r
Cell state ranks
CD4+ Tcm
Treg
CD8+ Teff (KLRC1)
NK (cycling)
CD8+ T cell (MHC II)
Monocyte (FCGR3A)
Pericyte (cycling)
c
Treg IFNγ response signature score
Fraction of IFNG+ CD8+ T cells
Pericyte (cycling) IFNγ response signature score
Fraction of IFNG+ CD8+ T cells

Supplementary Fig. 4. Interferon-γ expression by CD8$^+$ T cells is correlated to interferon-response signature in multiple cell types in the esophageal mucosa. **a.** CD8$^+$ T cells, T_H1, and circulating *FCGR3A*$^+$ NK cells express interferon-γ (*IFNG*). Distribution of expression (*y* axis) of IFN-γ (first row), interferon-γ receptor 1 (second row, *IFNGR1*), interferon-γ receptor 2 (third row, *IFNGR2*), IFN-γ response signature (fourth row), IFN-α response signature (fifth row), and a random gene signature (sixth row, with the same number of genes as in the IFN-γ response signature) across the cell subsets in our atlas (*x* axis). **b,c.** Proportion of *IFNG*$^+$ CD8$^+$ T cells is positively correlated across samples to mean IFN-γ response signature for many cell subsets. **b.** Pearson correlation coefficients (R) between the proportion of *IFNG*$^+$ CD8$^+$ T cells (of all CD8$^+$ T cells) and the average interferon-γ response gene signature score for each cell type (*x* axis, rank ordered by Pearson's R) across samples. **c.** *IFNG*$^+$ CD8$^+$ T cell proportions (of all CD8$^+$ T cells, *x* axis) and the mean interferon-γ response gene signature score (*y* axis) for T_{regs} (left) or cycling pericytes (right) in each sample (dot). Only samples with at least one detected CD8$^+$ T cell and T_{reg} / cycling pericyte are shown. Blue lines: linear regression; gray area: 95% confidence intervals. Source data are provided as a Source Data file.

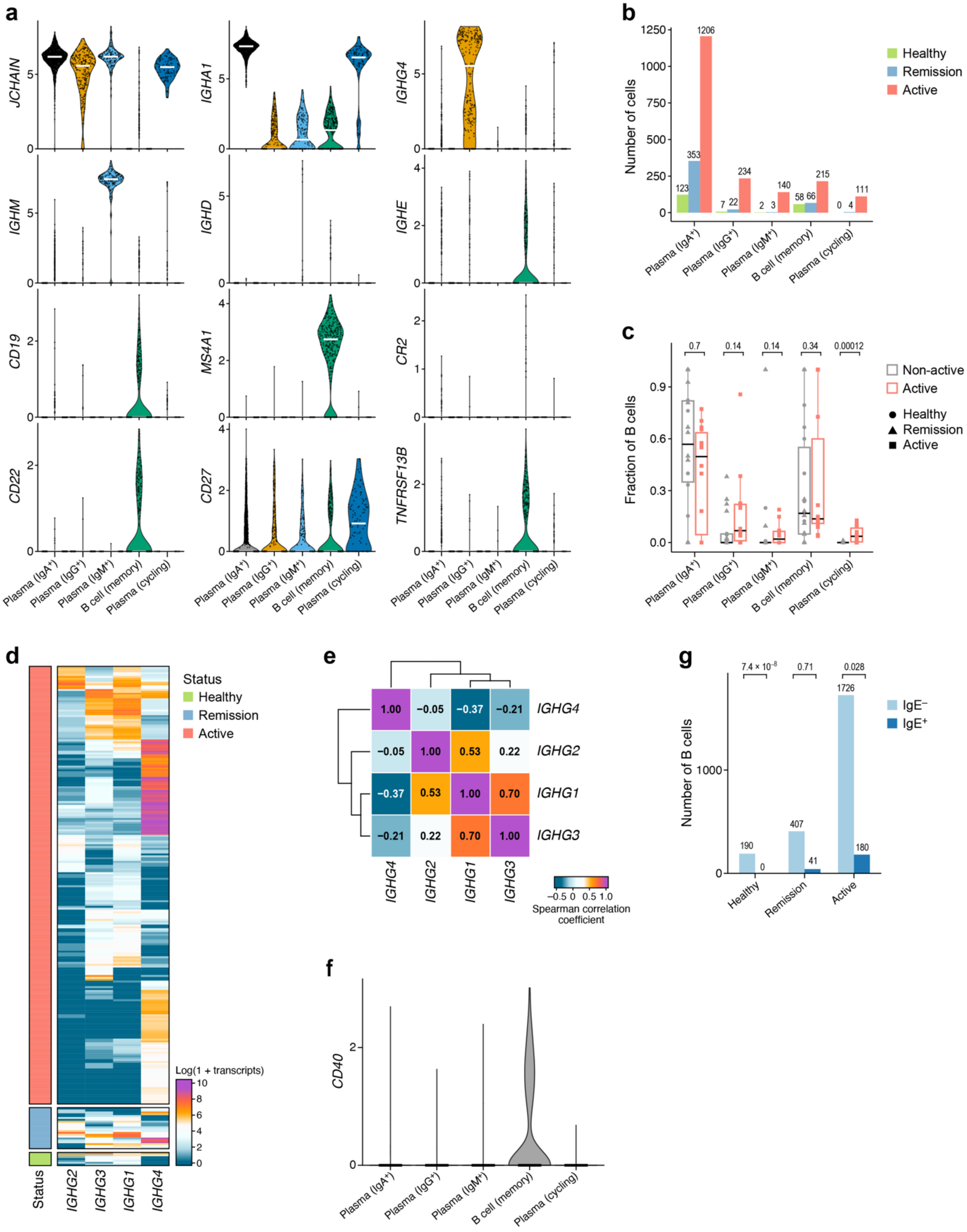

a
JCHAIN
IGHA1
IGHG4
IGHM
IGHD
IGHE
CD19
MS4A1
CR2
CD22
CD27
TNFRSF13B
Plasma (IgA+)
Plasma (IgG+)
Plasma (IgM+)
B cell (memory)
Plasma (cycling)
b
Number of cells
Healthy
Remission
Active
123
353
1206
7
22
234
2
3
140
58
66
215
0
4
111
c
Fraction of B cells
0.7
0.14
0.14
0.34
0.00012
Non-active
Active
Healthy
Remission
Active
d
Status
Healthy
Remission
Active
Log(1 + transcripts)
IGHG2
IGHG3
IGHG1
IGHG4
e
IGHG4
IGHG2
IGHG1
IGHG3
Spearman correlation coefficient
f
CD40
g
Number of B cells
7.4 × 10^-8
0.71
0.028
IgE−
IgE+
190
0
407
41
1726
180
Healthy
Remission
Active

Supplementary Fig. 5. Expansion of diverse plasma B cells in active EoE. a. Plasma and memory B cell marker expression in esophageal B cell subtypes. Distribution of expression (*y* axis, $\log_{10}$(TP10K+1)) of key marker genes in cells (dots) of each B cell subset (*x* axis). **b,c.** B cell expansion in EoE. **b.** Number of cells (*y* axis, label on top) of different B cell subsets (*x* axis) in each condition (colored bar). **c.** Distributions of cell type proportions (*y* axis) of each B cell subset (*x* axis) out of all B cells in active EoE (red squares, *n* = 14) *vs.* remission (gray triangles, *n* = 11) and healthy (gray circles, *n* = 12) biopsies. Boxplots: medians and interquartile ranges (IQR). Whiskers: lowest datum within 1.5 IQR of the lower quartile and highest datum within 1.5 IQR of the upper quartile. Top: FDR (two-tailed Wald test). **d,e.** IgG gene expression in IgG^+ plasma B cells. **d.** Expression (log (1+transcripts)) of each IgG gene (columns) in each cell (row) from each sample type (bar on left). **e.** Spearman's rank correlation coefficients (color bar) between expression profiles of IgG genes (rows, columns) across IgG^+ plasma B cells. **f.** *CD40* is highly expressed in memory B cells. Distribution of expression (*y* axis, $\log_{10}$(TP10K+1)) of *CD40* in each B cell subset (*x* axis). **g**. IgE detected in B cells from patients in either active disease or remission but absent in B cells from healthy participants. Number of B cells (*y* axis) where IgE transcript is detected (IgE^+, dark blue) or not (IgE^-, light blue) in each condition (*x* axis). Top: FDR (two-tailed Fisher's exact test). Violin plot widths: Gaussian kernel density estimation of data with default parameters, scaled to a maximum of 1; white horizontal segment: median. Source data are provided as a Source Data file.

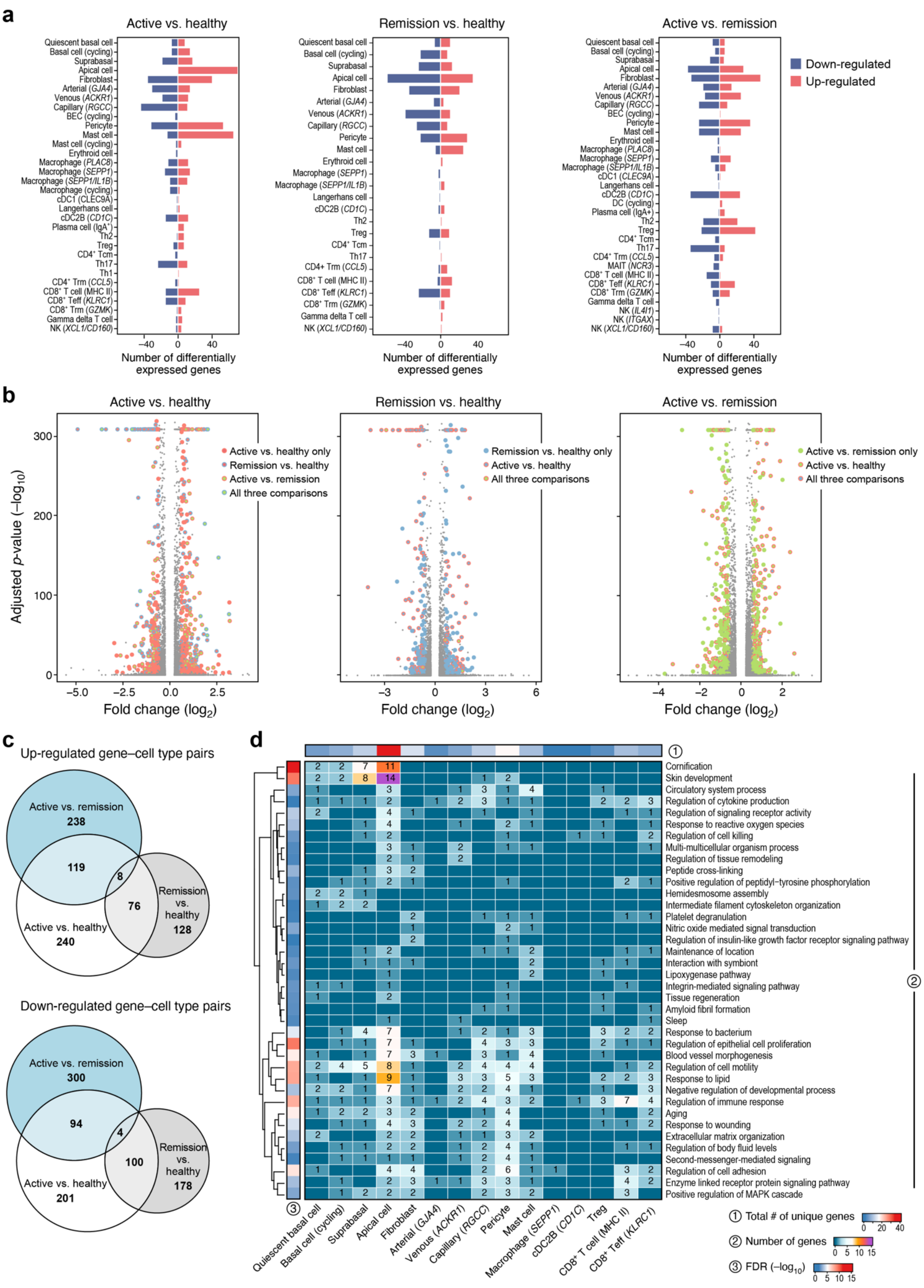
a
Active vs. healthy
Remission vs. healthy
Active vs. remission
Down-regulated
Up-regulated
Quiescent basal cell
Basal cell (cycling)
Suprabasal
Apical cell
Fibroblast
Arterial (GJA4)
Venous (ACKR1)
Capillary (RGCC)
BEC (cycling)
Pericyte
Mast cell
Mast cell (cycling)
Erythroid cell
Macrophage (PLAC8)
Macrophage (SEPP1)
Macrophage (SEPP1/IL1B)
Macrophage (cycling)
cDC1 (CLEC9A)
Langerhans cell
cDC2B (CD1C)
DC (cycling)
Plasma cell (IgA+)
Th2
Treg
CD4+ Tcm
Th17
Th1
CD4+ Trm (CCL5)
MAIT (NCR3)
CD8+ T cell (MHC II)
CD8+ Teff (KLRC1)
CD8+ Trm (GZMK)
Gamma delta T cell
NK (IL4I1)
NK (ITGAX)
NK (XCL1/CD160)
−40
0
40
Number of differentially expressed genes
b
Adjusted p-value (−log10)
Fold change (log2)
Active vs. healthy only
Remission vs. healthy
Active vs. remission
All three comparisons
Remission vs. healthy only
Active vs. healthy
Active vs. remission only
c
Up-regulated gene–cell type pairs
Active vs. remission
238
119
8
76
Remission vs. healthy
128
Active vs. healthy
240
Down-regulated gene–cell type pairs
Active vs. remission
300
94
4
100
Remission vs. healthy
178
Active vs. healthy
201
d
Cornification
Skin development
Circulatory system process
Regulation of cytokine production
Regulation of signaling receptor activity
Response to reactive oxygen species
Regulation of cell killing
Multi-multicellular organism process
Regulation of tissue remodeling
Peptide cross-linking
Positive regulation of peptidyl−tyrosine phosphorylation
Hemidesmosome assembly
Intermediate filament cytoskeleton organization
Platelet degranulation
Nitric oxide mediated signal transduction
Regulation of insulin-like growth factor receptor signaling pathway
Maintenance of location
Interaction with symbiont
Lipoxygenase pathway
Integrin-mediated signaling pathway
Tissue regeneration
Amyloid fibril formation
Sleep
Response to bacterium
Regulation of epithelial cell proliferation
Blood vessel morphogenesis
Regulation of cell motility
Response to lipid
Negative regulation of developmental process
Regulation of immune response
Aging
Response to wounding
Extracellular matrix organization
Regulation of body fluid levels
Second-messenger-mediated signaling
Regulation of cell adhesion
Enzyme linked receptor protein signaling pathway
Positive regulation of MAPK cascade
Quiescent basal cell
Basal cell (cycling)
Suprabasal
Apical cell
Fibroblast
Arterial (GJA4)
Venous (ACKR1)
Capillary (RGCC)
Pericyte
Mast cell
Macrophage (SEPP1)
cDC2B (CD1C)
Treg
CD8+ T cell (MHC II)
CD8+ Teff (KLRC1)
① Total # of unique genes
② Number of genes
③ FDR (−log10)

Supplementary Fig. 6. Changes in cell intrinsic expression programs in EoE. **a.** Number of differentially expressed genes (*x* axis) in each cell type (*y* axis) from pairwise comparisons between active EoE and healthy (left), remission and healthy (center), and active EoE and remission (right). **b**. Significance (*y* axis ($-\log_{10}$(*p*-value), two-tailed likelihood-ratio tests of logistic regression coefficients, Bonferroni corrected) of differential expression (fold change, *x* axis) for each gene–cell type pair (dot) between each pair of conditions. Red: >1.5-fold change, adjusted $p < 0.001$. Gene–cell pairs (dots) that are also differentially expressed in another comparison are color-coded by inner point and outer ring colors (color legend). **c.** Venn diagrams of differentially expressed gene-cell type pairs from the pairwise comparisons in **b**. **d.** Enrichment ($-\log_{10}$(FDR), one-tailed hypergeometric tests) of Gene Ontology biological process terms (rows) in genes differentially expressed (numbers) both between active EoE and health and between remission and health, in each cell type (columns). Source data are provided as a Source Data file.

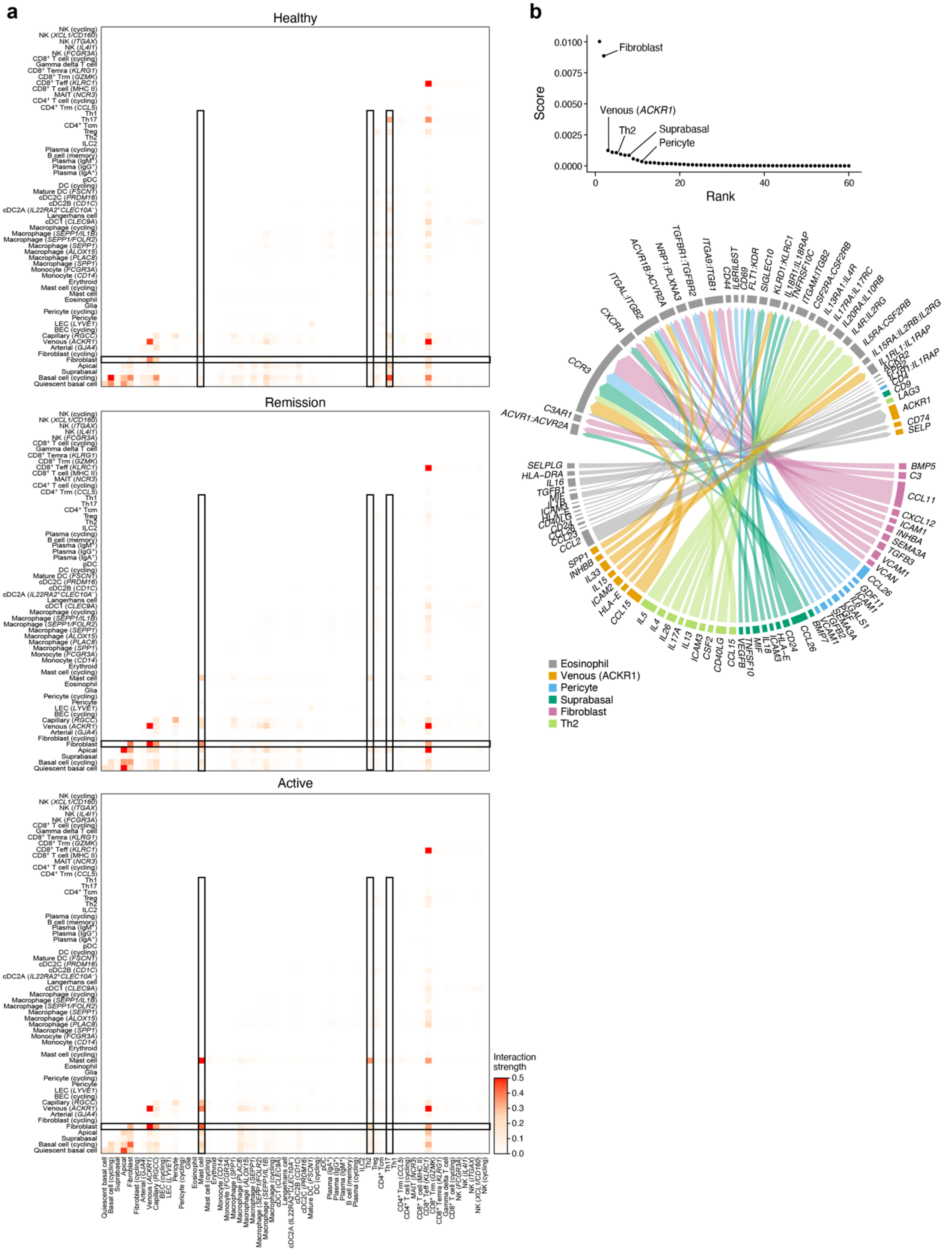
a
Healthy
Remission
Active
Interaction strength
0.5
0.4
0.3
0.2
0.1
0.0
b
Score
Rank
0.0100
0.0075
0.0050
0.0025
0.0000
0
20
40
60
Fibroblast
Venous (ACKR1)
Th2
Suprabasal
Pericyte
Eosinophil
Venous (ACKR1)
Pericyte
Suprabasal
Fibroblast
Th2

Supplementary Fig. 7. Cell–cell communication changes by disease status and eosinophil recruitment. **a.** Changes in cell interactions between conditions. Putative interaction strength (color bar) between each pair of cells (rows, columns) in healthy (top), remission (middle), and active EoE (bottom) samples. Boxes highlight interactions between mast, T_H2, and T_H17 cells and fibroblasts that change in strength between conditions. **b.** Prominent interactions with eosinophils. Top: Interaction scores (*y* axis) between eosinophils and each of the 60 prevalent cell subsets (*x* axis), rank ordered by scores. Bottom: Ligand–receptor pairs expressed between eosinophils (grey) and T_H2, venous endothelial cells, suprabasal cells, fibroblasts, and pericytes (outer ring color coded by expressing cell subset and labeled by expressed gene names). Sector width: total strength of sector connecting to other sectors. Edges width between ligands and receptors are proportional to the strengths of the interactions and are colored by the ligand-expressing cell type. Source data are provided as a Source Data file.

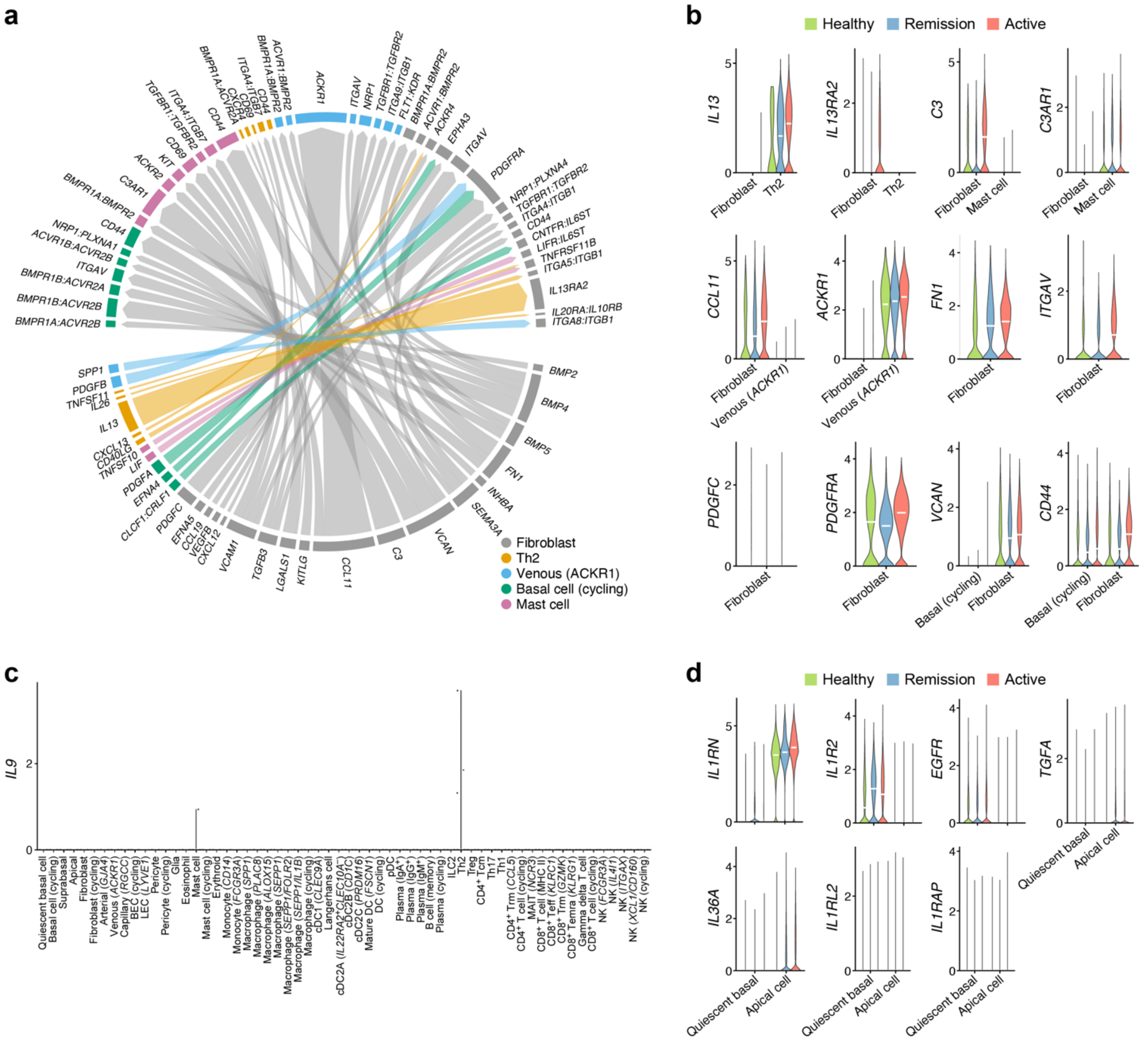
a
b
Healthy
Remission
Active
Fibroblast
Th2
Venous (ACKR1)
Basal cell (cycling)
Mast cell
c
d

Supplementary Fig. 8. Cellular interactions that may mediate EoE. a. Prominent interactions with fibroblasts. Ligand–receptor pairs expressed between fibroblasts (grey) and T_H2, mast cells, cycling basal cells, and venous endothelial cells (outer ring color coded by expressing cell subset and labeled by expressed gene name). Sector width: total strength of sector connecting to other sectors. Edges width between ligands and receptors are proportional to the strengths of the interactions and are colored by the ligand-expressing cell type. **b.** Key ligand–receptor interactions with fibroblasts. Distribution of expression (*y* axis, $\log_{10}$(TP10K+1)) in the relevant cell subsets (*x* axis) in each condition (color code) of genes encoding top ligand–receptor interacting pairs between fibroblasts and T_H2 cells (*IL13–IL13RA2*), mast cells (*C3–C3RA1*), venous endothelial cells (*CCL11–ACKR1*), fibroblasts (*PDGFC–PDGFRA, FN1–ITGAV*), and cycling basal cells (*VCAN–CD44*). **c.** T_H2 cells from some active EoE patients express the mast cell growth factor IL-9. Distribution of expression (*y* axis, $\log_{10}$(TP10K+1)) of *IL9* across cells (dots) from each cell subset (*x* axis). **d.** Key ligand–receptor interactions between quiescent basal cells and apical cells. Distribution of expression (*y* axis, $\log_{10}$(TP10K+1)) in quiescent basal and apical cell subsets (*x* axis) of genes encoding top putative interacting ligand–receptor pairs (*IL1RN–IL1R2*, *TGFA–EGFR*, *IL36A–IL1RL2*/*IL1RAP*). Violin plot widths: Gaussian kernel density estimation of data with default parameters, scaled to a maximum of 1; white horizontal segment: median. Source data are provided as a Source Data file.

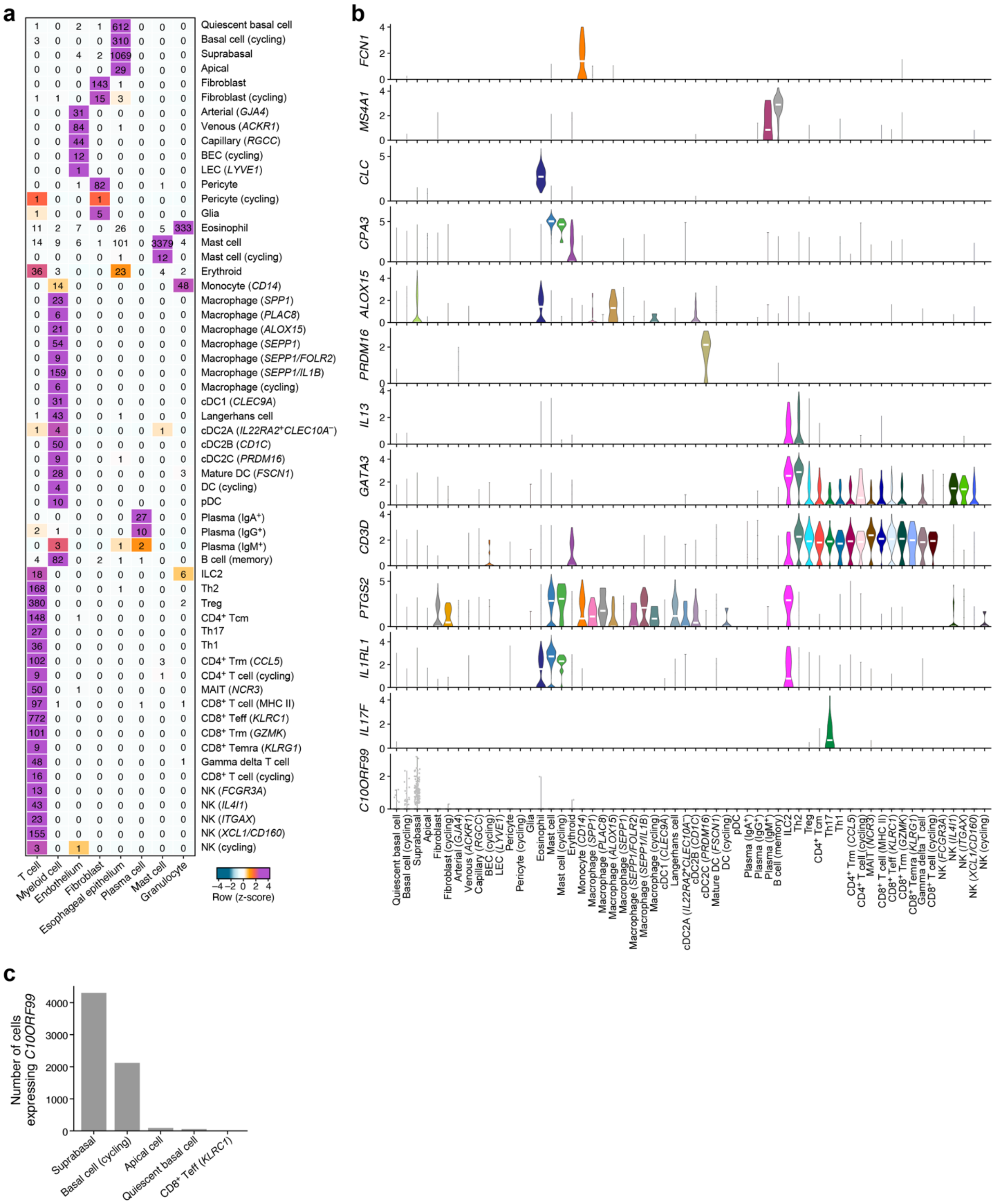
a
b
c
FCN1
MS4A1
CLC
CPA3
ALOX15
PRDM16
IL13
GATA3
CD3D
PTGS2
IL1RL1
IL17F
C10ORF99
T cell
Myeloid cell
Endothelium
Fibroblast
Esophageal epithelium
Plasma cell
Mast cell
Granulocyte
Row (z-score)
Number of cells expressing C10ORF99
Suprabasal
Basal cell (cycling)
Apical cell
Quiescent basal cell
CD8+ Teff (KLRC1)

Supplementary Fig. 9. Using the esophageal atlas as a reference. a,b. Mapping smaller scRNA-seq EoE data sets[3] to the esophageal cell atlas refines prior cell annotations. **a.** Number of cells (color bar, row Z-score, numbers) from each annotated subset in a published dataset[3] (columns) assigned to the refined subsets in our esophageal atlas (rows). **b.** Distribution of expression (*y* axis, $\log_{10}$(TP10K+1)) of key marker genes in cells of the published cell profiles (dots) of Ref.[3] reannotated and reassigned to subsets (*x* axis) based on our atlas, including cells misannotated (monocytes, memory B cells) or missed (*ALOX15*$^+$ macrophages, *PRDM6*$^+$ cDC2Cs, ILC2s) in the original study. **c.** Suprabasal cells are a major source of *GPR15* ligand (*C10ORF99*) expression in both datasets. Number of cells expressing *C10ORF99* (*y* axis) in each cell subset (*x* axis) from Ref.[3] after reannotation by our atlas (see also last row in **b**). Violin plot widths: Gaussian kernel density estimation of data with default parameters, scaled to a maximum of 1; white horizontal segment: median. Source data are provided as a Source Data file.

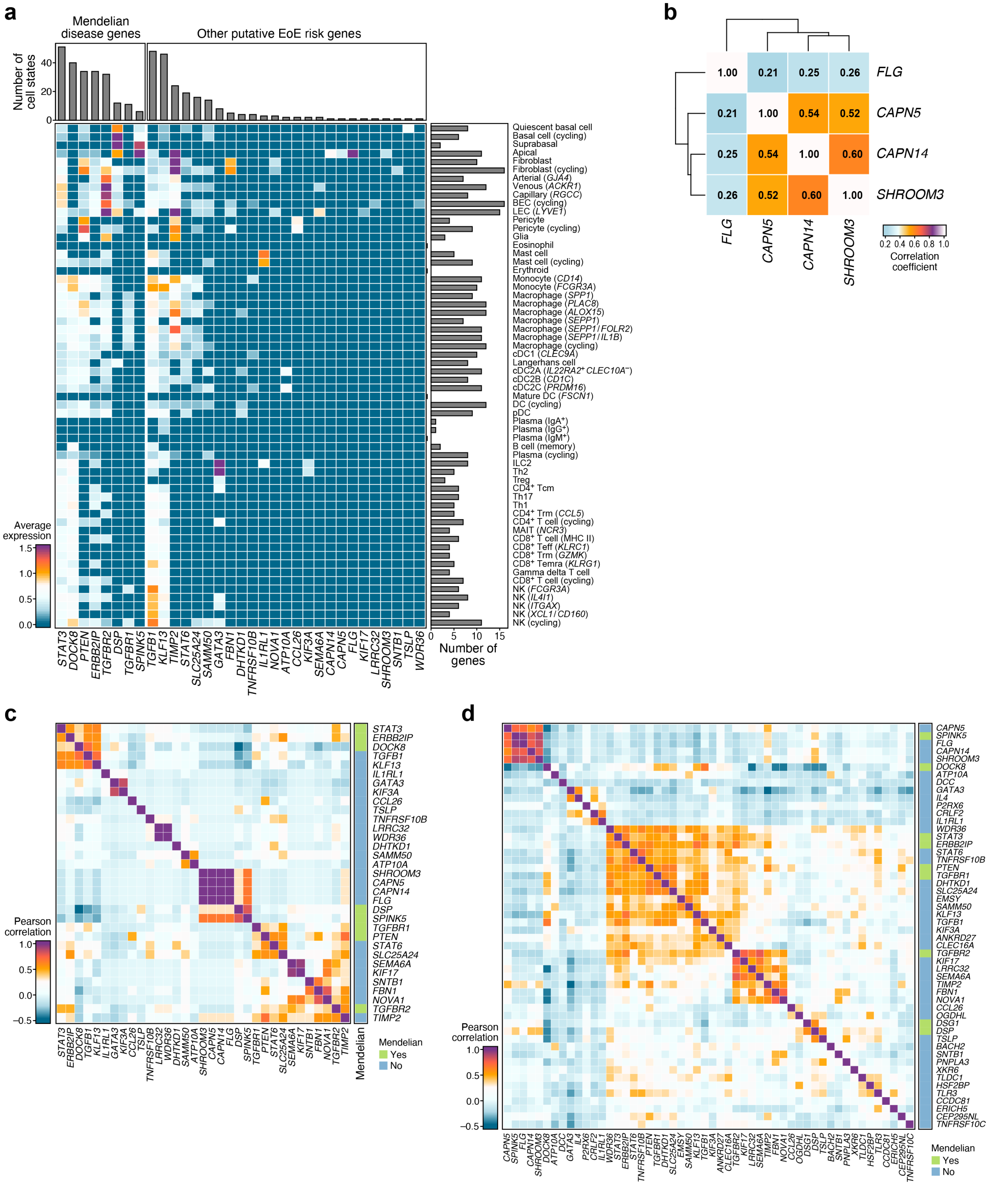
a
Mendelian disease genes
Other putative EoE risk genes
Number of cell states
Average expression
STAT3 DOCK8 PTEN ERBB2IP TGFBR2 DSP TGFBR1 SPINK5 TGFB1 KLF13 TIMP2 STAT6 SLC25A24 SAMM50 GATA3 FBN1 DHTKD1 TNFRSF10B IL1RL1 NOVA1 ATP10A CCL26 KIF3A SEMA6A CAPN14 CAPN5 FLG KIF17 LRRC32 SHROOM3 SNTB1 TSLP WDR36
Quiescent basal cell
Basal cell (cycling)
Suprabasal
Apical
Fibroblast
Fibroblast (cycling)
Arterial (GJA4)
Venous (ACKR1)
Capillary (RGCC)
BEC (cycling)
LEC (LYVE1)
Pericyte
Pericyte (cycling)
Glia
Eosinophil
Mast cell
Mast cell (cycling)
Erythroid
Monocyte (CD14)
Monocyte (FCGR3A)
Macrophage (SPP1)
Macrophage (PLAC8)
Macrophage (ALOX15)
Macrophage (SEPP1)
Macrophage (SEPP1/FOLR2)
Macrophage (SEPP1/IL1B)
Macrophage (cycling)
cDC1 (CLEC9A)
Langerhans cell
cDC2A (IL22RA2⁺ CLEC10A⁻)
cDC2B (CD1C)
cDC2C (PRDM16)
Mature DC (FSCN1)
DC (cycling)
pDC
Plasma (IgA⁺)
Plasma (IgG⁺)
Plasma (IgM⁺)
B cell (memory)
Plasma (cycling)
ILC2
Th2
Treg
CD4⁺ Tcm
Th17
Th1
CD4⁺ Trm (CCL5)
CD4⁺ T cell (cycling)
MAIT (NCR3)
CD8⁺ T cell (MHC II)
CD8⁺ Teff (KLRC1)
CD8⁺ Trm (GZMK)
CD8⁺ Temra (KLRG1)
Gamma delta T cell
CD8⁺ T cell (cycling)
NK (FCGR3A)
NK (IL4I1)
NK (ITGAX)
NK (XCL1/CD160)
NK (cycling)
Number of genes
b
FLG CAPN5 CAPN14 SHROOM3
Correlation coefficient
c
Pearson correlation
STAT3 ERBB2IP DOCK8 TGFB1 KLF13 IL1RL1 GATA3 KIF3A CCL26 TSLP TNFRSF10B LRRC32 WDR36 DHTKD1 SAMM50 ATP10A SHROOM3 CAPN5 CAPN14 FLG DSP SPINK5 TGFBR1 PTEN STAT6 SLC25A24 SEMA6A KIF17 SNTB1 FBN1 NOVA1 TGFBR2 TIMP2
Mendelian
Yes
No
d
Pearson correlation
CAPN5 SPINK5 FLG CAPN14 SHROOM3 DOCK8 ATP10A DCC GATA3 IL4 P2RX6 CRLF2 IL1RL1 WDR36 STAT3 ERBB2IP STAT6 TNFRSF10B PTEN TGFBR1 DHTKD1 SLC25A24 EMSY SAMM50 KLF13 TGFB1 KIF3A ANKRD27 CLEC16A TGFBR2 KIF17 LRRC32 SEMA6A TIMP2 FBN1 NOVA1 CCL26 OGDHL DSG1 DSP TSLP BACH2 SNTB1 PNPLA3 XKR6 TLDC1 HSF2BP TLR3 CCDC81 ERICH5 CEP295NL TNFRSF10C
Mendelian
Yes
No

Supplementary Fig. 10. EoE risk gene expression across the esophageal cell atlas. **a.** Cell type specific expression of Mendelian and common EoE risk genes. Mean expression ($\log_{10}$(TP10K+1)) of Mendelian genes for EoE-associated diseases (columns, left) and of genes with common variants associated with EoE risk (columns, right) in each cell subset in the atlas (rows). Top bar plot: number of cell subsets (*y* axis) expressing each gene (*x* axis). Right bar plot: number of EoE genes (*x* axis) expressed in each cell subset (*y* axis). Expression of genes detected in <25% of cells in a subset is shown as zero. **b–d**. Risk genes co-vary by expression in modules within and across cell types. **b.** Spearman's rank correlation coefficients across apical cells between expression profiles of apical cell-specific risk genes (rows, columns). **c,d.** Pearson correlation coefficients (color bar) between mean expression profiles (**c**, mean profiles as in (**a**) and only genes in (**a**) are used) or between pseudobulk expression of all the 52 (**d**) EoE Mendelian (green) and common (blue) risk genes (rows, columns), clustered with complete linkage (**c**) or Ward.D2 linkage (**d**) hierarchical clustering. Source data are provided as a Source Data file.

Supplementary Note 1

Our analysis identified all 8 subsets of cells in the human stomach oxyntic gland[4] (**Supplementary Fig. 2d**). These cells include chief cells (*PGA3*/*4*/*5*), delta cells (*SST*, *TPPP3*, and *RBP4*), foveolar cells (*TFF1*, *MUC5AC*, and *GKZ2*), gastrin cells (*GAST*, *CHGB*, and *CES1*), ghrelin cells (*GHRL*, *APOA1*, and *TM4SF4*), mucous neck cells (*MUC6*, *LYZ*, and *TFF2*), parietal cells (*ATP4A*, *ATP4B*, and *GIF*), and enterochromaffin-like cells (*HDC*, *TPH1*, and *LHX5*). These cells were rare and almost all of them were from the distal sample of patient E1343 and from patient E1503.

Four other rare cell types also showed patient specificity, including alveolar type II epithelial cells (*SFTPA1*, *SFTPB*, and *SCGB3A2*), Paneth cells (*PLA2G2A*, *DEFA6*, and *PRSS2*), gland duct cells (*KRT23*, *ALDH1A3*, and *MMP7*, showing patient specificity in a recent study[5]), and smooth muscle cells (SMCs) (*DES*, *ACTG2*, and *PLN*). We removed all these rare and patient-specific cells from further analysis.

In general, relative to other cell types, tissue eosinophils demonstrated globally lower levels of transcriptional activity. We detected a median of only 361 genes per cell for eosinophils. Many eosinophils may not have passed our initial filtering because of the small number of genes or unique molecular identifiers (UMIs) captured by the droplet-based 10x Chromium platform. To recover the lost eosinophils, we extracted candidate eosinophils expressing *CLC* and performed a clustering analysis to filter likely non-eosinophils by canonical marker genes. All together, we obtained an extra 3,150 eosinophil candidates (3,275 eosinophil candidates in total).

Supplementary References

1. Kowalczyk, M. S. *et al.* Single-cell RNA-seq reveals changes in cell cycle and differentiation programs upon aging of hematopoietic stem cells. *Genome Res.* **25**, 1860–1872 (2015).
2. Ferrer-Torres, D. *et al.* Mapping the adult human esophagus in vivo and in vitro. *Development* **149**, dev200614 (2022).
3. Morgan, D. M. *et al.* Clonally expanded, GPR15-expressing pathogenic effector TH2 cells are associated with eosinophilic esophagitis. *Sci. Immunol.* **6**, eabi5586 (2021).
4. Busslinger, G. A. *et al.* Human gastrointestinal epithelia of the esophagus, stomach, and duodenum resolved at single-cell resolution. *Cell Rep.* **34**, 108819 (2021).
5. Madissoon, E. *et al.* scRNA-seq assessment of the human lung, spleen, and esophagus tissue stability after cold preservation. *Genome Biol.* **21**, (2020).
