# Supplementary material for: An esophagus cell atlas reveals dynamic rewiring during active eosinophilic esophagitis and remission
Source: Nat Commun. 2024 Apr 18;15:3344. doi: 10.1038/s41467-024-47647-0 (PMC11026436; doi:10.1038/s41467-024-47647-0)
Supplement: Supplementary file 9 — Reporting Summary [file 41467_2024_47647_MOESM9_ESM.pdf]

nature portfolio

Corresponding author(s): Aviv Regev and Ramnik Xavier

Last updated by author(s): Jan 3, 2024

Reporting Summary

Nature Portfolio wishes to improve the reproducibility of the work that we publish. This form provides structure for consistency and transparency in reporting. For further information on Nature Portfolio policies, see our Editorial Policies and the Editorial Policy Checklist.

Statistics

For all statistical analyses, confirm that the following items are present in the figure legend, table legend, main text, or Methods section.

n/a	Confirmed	
☐	☒	The exact sample size (n) for each experimental group/condition, given as a discrete number and unit of measurement
☐	☒	A statement on whether measurements were taken from distinct samples or whether the same sample was measured repeatedly
☐	☒	The statistical test(s) used AND whether they are one- or two-sided<br>*Only common tests should be described solely by name; describe more complex techniques in the Methods section.*
☐	☒	A description of all covariates tested
☐	☒	A description of any assumptions or corrections, such as tests of normality and adjustment for multiple comparisons
☐	☒	A full description of the statistical parameters including central tendency (e.g. means) or other basic estimates (e.g. regression coefficient) AND variation (e.g. standard deviation) or associated estimates of uncertainty (e.g. confidence intervals)
☐	☒	For null hypothesis testing, the test statistic (e.g. F, t, r) with confidence intervals, effect sizes, degrees of freedom and P value noted<br>*Give P values as exact values whenever suitable.*
☒	☐	For Bayesian analysis, information on the choice of priors and Markov chain Monte Carlo settings
☒	☐	For hierarchical and complex designs, identification of the appropriate level for tests and full reporting of outcomes
☐	☒	Estimates of effect sizes (e.g. Cohen's d, Pearson's r), indicating how they were calculated

Our web collection on statistics for biologists contains articles on many of the points above.

Software and code

Policy information about availability of computer code

Data collection	Sequenced data were shared with us via FTP and were used for read demultiplexing and gene-cell expression matrix generation.
Data analysis	CellRanger-2.1.1 (for 10x Chromium v2 chemistry data), CellRanger-3.1.0 (for Chromium v3 chemistry data) and CellRanger-5.0.0 (for Chromium v3 chemistry dual index data) were used for read demultiplexing, alignment to the human GRCh38 genome (from CellRanger refdata v1.2.0), and unique molecular identifier (UMI) counting and collapsing. scPhere v0.1.0 was used for dimension reduction and visualization, at the same time correcting for batch effects. Clustering analyzing on scPhere results was performed using Seurat v4.1.1. Subclustering to detect fine-grained cell subsets were based on Seurat v4.1.1 and DensityCut v0.01. Logistic regression was used for differential expression analysis, taking both the log2-transformed total number of detected genes in each cell, 10x Chromium library version, steroid treatment, and spatial location of biopsy as covariates, using the Seurat implementation for differential expression analysis. All statistical tests were based on R v4.2.0. The STRINGdb Bioconductor package (using STRING v11) was used for Gene Ontology enrichment analysis with the `get_enrichment' function for enrichment analysis, and all the genes in CellRanger refdata v1.2.0, after removing mitochondrial genes and ribosome protein coding genes (i.e., RPS* and RPL* genes), were used as the background gene list. Customized scripts can be found in this epo: https://github.com/Ding-Group/eoe

For manuscripts utilizing custom algorithms or software that are central to the research but not yet described in published literature, software must be made available to editors and reviewers. We strongly encourage code deposition in a community repository (e.g. GitHub). See the Nature Portfolio guidelines for submitting code & software for further information.

Data

Policy information about availability of data

All manuscripts must include a data availability statement. This statement should provide the following information, where applicable:

- Accession codes, unique identifiers, or web links for publicly available datasets
- A description of any restrictions on data availability
- For clinical datasets or third party data, please ensure that the statement adheres to our policy

Processed data are available from the Single-Cell Portal with accession number SCP1242: https://singlecell.broadinstitute.org/single_cell/study/SCP1242, and the raw human sequencing data are available from the controlled access DUOS system with accession number DUOS-000XXX.

We directly downloaded the publicly available 1,088 T cell data from GEO (GSE126250). The pediatric EoE data were downloaded from GEO (GSE175930).

The GBM data can be downloaded from Brain Immune Atlas (https://www.brainimmuneatlas.org/download.php). The colon dataset is from this website (https://www.gutcellatlas.org/). The cross-tissue immune cells can be downloaded from this website (https://www.tissueimmunecellatlas.org/). The Tabula Sapiens data can be downloaded from this website (https://tabula-sapiens-portal.ds.czbiohub.org/).

The GRCh38 human reference 1.2.0 were downloaded from 10x Genomics (https://www.10xgenomics.com/support/software/cell-ranger/downloads) and can be prepared according to these instructions (https://www.10xgenomics.com/support/software/cell-ranger/downloads/cr-ref-build-steps).

Research involving human participants, their data, or biological material

Policy information about studies with human participants or human data. See also policy information about sex, gender (identity/presentation), and sexual orientation and race, ethnicity and racism.

Reporting on sex and gender	Sex information based on clinical records is reported for each individual in Supplementary Data 1. Recruitment was performed independently of sex, and the total cohort consists of 11 males and 11 females. Given the limited number of subjects within each phenotype, no sex specific analyses were performed in this study.
Reporting on race, ethnicity, or other socially relevant groupings	Race and ethnicity information was not collected as part of these studies.
Population characteristics	Our patient cohort consists of 22 adult donors (> 18 year old, 11 female and 11 male), with 7 control participants, 7 remission EoE patients, and 8 active EoE patients. Patients have undergone different treatments (PPI, food elimination, and steroid). Covariate-relevant information is provided in Supplementary Data 1.
Recruitment	Participants were recruited during visits at Massachusetts General Hospital. We did not notice any bias in participant recruitment.
Ethics oversight	Mass General Brigham Institutional Review Board

Note that full information on the approval of the study protocol must also be provided in the manuscript.

Field-specific reporting

Please select the one below that is the best fit for your research. If you are not sure, read the appropriate sections before making your selection.

☒ Life sciences ☐ Behavioural & social sciences ☐ Ecological, evolutionary & environmental sciences

For a reference copy of the document with all sections, see nature.com/documents/nr-reporting-summary-flat.pdf

Life sciences study design

All studies must disclose on these points even when the disclosure is negative.

Sample size	We chose the number of cells to profile based on the cell capture rate of the 10x Chromium platform, the ratios of different cell types in the studied tissue, and the costs. With 400,000 cells, we were able to recover at least 20 cells for each cell type with probability greater than 0.99 (https://satijalab.org/howmanycells) given an estimated 80 cell types.
Data exclusions	All data and samples were included. Low quality cells were excluded as described in the Methods, briefly, cells with <500 UMI or >25% mitochondrial reads (v2 chemistry) or >40% mitochondrial reads (v3 chemistry) were excluded from downstream analyses but are present in the raw datasets provided.
Replication	The cell types have been detected in different donors, suggesting that the results are reproducible. In total, we collected cells from 37 biopsies of 22 individuals.
Randomization	Not relevant to our study as all samples were processed following the same protocol and no interventions were performed.

Blinding	Not relevant to our study as all samples were processed following the same protocol and no interventions were performed. Disease phenotype was a key element of the analysis.

Reporting for specific materials, systems and methods

We require information from authors about some types of materials, experimental systems and methods used in many studies. Here, indicate whether each material, system or method listed is relevant to your study. If you are not sure if a list item applies to your research, read the appropriate section before selecting a response.

Materials & experimental systems

n/a	Involved in the study
☒	☐ Antibodies
☒	☐ Eukaryotic cell lines
☒	☐ Palaeontology and archaeology
☒	☐ Animals and other organisms
☒	☐ Clinical data
☒	☐ Dual use research of concern
☒	☐ Plants

Methods

n/a	Involved in the study
☒	☐ ChIP-seq
☒	☐ Flow cytometry
☒	☐ MRI-based neuroimaging

Plants

Seed stocks	n/a
Novel plant genotypes	n/a
Authentication	n/a
